# 178. Comparing the Incidence of Multidrug Resistant Bacteremia, Fungemia and Hospital-acquired *Clostridioides difficile* Infection in COVID-19 Versus Non-COVID-19 Patients: a Single Hospital, One-year Observational Study in New York City

**DOI:** 10.1093/ofid/ofab466.380

**Published:** 2021-12-04

**Authors:** Chia-Yu Chiu, Amara Sarwal, Michael Widjaja, Addi Feinstein

**Affiliations:** 1 Department of Infectious Disease, The University of Texas Health Science Center at Houston, Houston, Texas; 2 Department of Nephrology, University of Utah, Salt Lake City, Utah; 3 Lincoln Medical Center, New York City, New York

## Abstract

This abstract has been withdrawn.

